# The effect of online social evaluation on mood and cognition in young people

**DOI:** 10.1038/s41598-022-24932-w

**Published:** 2022-12-05

**Authors:** Karina Grunewald, Jessica Deng, Jasmin Wertz, Susanne Schweizer

**Affiliations:** 1 University of New South Wales, Sydney, Australia; 2 University of Edinburgh, Edinburgh, UK; 3 University of Cambridge, Cambridge, UK

**Keywords:** Psychology, Human behaviour, Risk factors

## Abstract

Adolescence is characterised by increased peer interactions and heightened sensitivity to evaluation by peers. Increasingly, social interactions and evaluation happen in online contexts. Yet, little is known about the impact of online social interactions and evaluation on adolescent emotional and cognitive functioning. The present study examined the impact of online social evaluative threat on young people’s mood and learning and whether this varied as a function of known offline social risk and protective factors. 255 participants completed a perceptual learning task under online social evaluative threat and a perceptually-matched control condition. Participants were aged 11–30 years, to allow for the exploration of age differences in the impact of online social evaluative threat from adolescence to early adulthood. Participants reported a greater increase in negative mood (self-reported levels of stress, anxiety, and anhedonia), following social evaluative threat compared to the control condition. Heightened social rejection sensitivity (measured using the Online and Offline Social Sensitivity Scale) and lower perceived social support (measured using the Schuster Social Support Scale) were associated with elevated negative mood across the study. Social evaluative threat adversely impacted overall accuracy on the perceptual matching task, but not learning. These findings provide preliminary evidence that online social evaluative threat impacts adolescent mood and cognitive functioning.

## Introduction

### The effect of social evaluative threat on learning and well-being in young people

Adolescents (10–24 years)^1^ come of age within the context of changing social environments^2^. As a developmental period, adolescence is characterised by a shift in focus away from the immediate family towards interactions with peers and other members of society^3^. Social interactions during this period are often novel and less stable, playing a crucial role in adolescents’ developing sense of self, well-being and cognitive abilities^4^. Frequently, these interactions now occur in online contexts as adolescents spend a daily average of 6-h online for non-school purposes^5^, with the majority of this time spent on social media sites^6^. Yet, the impact of online social interactions on adolescent emotional and cognitive functioning remains little understood.

Work on the impact of social media use on emotional wellbeing has shown mixed results^7^. In her review of reviews on the association between social media use and wellbeing, Orben^7^ reports very small negative associations between wellbeing and social media use. Research extending beyond self-reported wellbeing shows that social media is associated with a range of both adaptive and maladaptive social and cognitive outcomes. For example, in the large (*N* > 12′000) British Understanding Society cohort, social media use has been prospectively associated with more positive peer relationships^8^. Cross-sectional data from the ethnically diverse US Adolescent Brain and Cognitive Development cohort (*N* > 11′000) has shown that self-reported social media use was associated with lower levels of cognitive functioning, though so were time spent watching TV and videos^9^. However, there was no association observed between cognitive functioning and social media use in a large, population representative sample of Austrian adolescents (*N* > 12′000)^10^. In a smaller sample, social media use was positively associated with cognitive functioning. The evidence on the impact of social media use on wellbeing and cognition then is mixed. Interestingly, a survey in 743 13–17 year-olds found that 31% reported social media having a mostly positive effect on their lives, whereas 24% of respondents thought it had a mostly negative impact^6^. Individual differences may help account for previous research’s mixed findings^7^. The present study therefore took an individual differences approach examining whether the impact of online social interactions on adolescent emotional and cognitive functioning varied as a function of interindividual differences in offline social risk and protective factors.

### Offline social risk and protective factors: Social rejection sensitivity and social support

Social relationships have been shown to foster resilience against emotional disorders during adolescence^11^. To form and maintain social relationships, adolescents become sensitive to various cues of social acceptance and rejection^12^. Excessive sensitivity to social rejection, however, is a known risk factor for emotional disorders^13^. Social rejection sensitivity refers to the tendency to expect social rejection by others^14^, be hypervigilant to social rejection cues and interpret ambiguous social situations negatively^15^. In addition to its association with adverse mental health outcomes, social rejection sensitivity may also negatively impact cognitive functioning and learning in adolescence. The attentional control theory posits that anxiety and uncertainty impair higher order cognitive functions and learning by reducing attentional control resources available for goal-relevant task processing^16^. The elevated levels of stress and anxiety experienced by individuals with higher social rejection sensitivity in the context of online social interactions may therefore lead to reduced attentional control and consequently poorer cognitive functioning.

In contrast, social support has been shown to be a protective factor in adolescents at risk for mental ill health^11,17,18^. While less is known about the effect of social support on adolescent cognitive functioning, evidence from the aging literature suggests it has a protective effect on cognition^19^. Conversely, the absence of social support, experienced through social isolation, is associated with poor mental health and reduced cognitive functioning in young people^20^ and related mid-life outcomes (incl., lower income)^21^.

### Risk and protective factors in digital interactions

Importantly, in the digital age, these risk and protective factors can operate anywhere and at any time. That is, the advent of online social interactions on mobile devices has eradicated non-social spaces. There are at least two potential implications. First, the perpetual potential for social rejection that ensues from online social interactions may negatively impact young people high in social rejection sensitivity. Second, the easy availability of social interactions may potentiate the beneficial effects of perceived social support by being more continuously available to booster resilience in young people.

Preliminary research shows that online social support is associated with increased well-being^22^ and contributes to improvements in low mood even after accounting for offline social support^23^. Cross-sectionally, both online and offline social rejection sensitivity are associated with symptoms of depressed mood^24^. The continuous threat of rejection may also impact other areas of functioning such as learning. Vocational and academic learning, especially homework and study, which now take place in the context of online social interactions, may be impacted differently as a function of individuals’ social rejection sensitivity and perceived social support. That is, building on attentional control theory, the anxiety caused by the continuous potential for social rejection may reduce the capacity to learn in individuals high on social rejection sensitivity, whereas young people with good social support may be less affected.

### The current study

This pre-registered study (https://osf.io/e8wyp) had two aims. The first was to investigate the effects of online social evaluative threat on adolescent mood and learning. The second aim was to explore whether these effects varied as a function of social risk (i.e., social rejection sensitivity) and protective (i.e. social support) factors, as well as self-reported mental health symptoms (primarily depressive symptoms) that are associated with these risk/protective factors.

To address these aims we designed a novel online social evaluative threat paradigm. Evaluative threat was evoked by asking participants to disclose personal information, which they were told would potentially be rated by peers. Under online social evaluative threat, participants then completed a perceptual learning task that has shown to be sensitive to social stress^25^. Performance on the task was compared to performance under no threat. 255 individuals aged 11–30 years completed the paradigm, which additionally allowed us to explore age-related differences across adolescence and early adulthood. Specifically, given research showing that adolescents are most sensitive to social rejection^12^ we predicted the impact of social rejection sensitivity in the current study would be greater in adolescents compared to young adults.

The study design allowed us to investigate the hypothesis that individuals with greater social rejection sensitivity would show increased negative mood (*Hypothesis 1a*) and poorer learning performance (*Hypothesis 1b*) following online social evaluative threat compared to the control condition, and the opposite would be shown by individuals with high levels of perceived social support. The magnitude of the impact of social evaluative threat (relative to control) on mood (*Hypothesis 2a*) and learning (*Hypothesis 2b*) would increase as a function of self-reported mental health problems. Given the proposed role of social rejection sensitivity in the onset and maintenance of depression^15^, we hypothesised that social rejection sensitivity would mediate the negative effect of depressive symptoms on mood and learning (*Hypothesis 3*).

## Methods

This study was approved by the University of New South Wales Human Research Ethics Executive Committee (HC number 200262) and was pre-registered prior to participant recruitment (https://osf.io/e8wyp). All research was performed in accordance with relevant regulations and performed in accordance with the Declaration of Helsinki. Informed consent was obtained from all participants.

### Participants

348 individuals aged 11–30 years were recruited through the University of New South Wales research participation system, social media advertisement, and the MQ participate page (a webpage created by a UK-based mental health research charity to advertise ongoing mental health research studies). A sample size of 181 was calculated by assuming a small-medium effect size (*f* = 0.12) of social threat^25^ to be detected in a model including the moderating effect of social rejection sensitivity, social support, and age at a power of 0.90 and a significance threshold of α = 0.05. While these calculations were conducted under the premise that participants would be completing the experiment in person (pre-pandemic), the present study had to be moved online due to restrictions caused by the COVID-19 pandemic. As our lab has previously found higher attrition with online experiments, we increased the sample size accordingly.

To participate in the study, individuals had to be fluent in English, be 11–30 years of age, and have no history of traumatic brain injury or a diagnosed learning disability. 93 participants were excluded based on these criteria as well as attention criteria (for details see supplementary materials). Participants aged 18 years and older provided informed consent prior to completing the study. Participants younger than 18 years provided informed assent and their parents provided informed consent for their child to participate. Individuals recruited through the university research participation system were compensated with course credit, whereas participants recruited through all other platforms received an AU$20 Coles (Australian supermarket chain) or Amazon voucher upon study completion. The final sample comprised 255 participants (Table 1).

**Table 1:** Demographic characteristics.

Demographics	*M* (*SD)*	*N (%)*
Age (years)	20.51 (4.27)	
**Gender**		
Female		158 (62)
Male		88 (35)
Other		5 (2)
Prefer not to say		0 (0)
N/A		4 (1)
**Ethnicity**		
Aboriginal/Torres Strait Islander		2 (1)
African		2 (1)
Asian		90 (35)
White		108 (42)
Hispanic		9 (4)
Mixed		19 (7)
Prefer not to say		8 (3)
Other		17 (7)
**Highest Education**		
Primary School		8 (3)
High School		100 (39)
Training		4 (2)
University		143 (56)
**Parental SES**		
Primary School		9.5 (4)
High School		57 (22)
Training		38 (15)
University		148 (58)
N/A		2.5 (1)

Training = Professional/Vocational Training; SES = social economic status (operationalised as parental level of education averaged for parent 1 and parent 2).

### Procedure

See Figure S1 for a schematic overview of the procedures. Following informed consent participants completed a baseline measure of mood, followed by self-report measures of demographics, mental health, social rejection sensitivity, and perceived social support. Next, participants completed the Raven’s Advanced Progressive Matrices and the affective digit span task (see supplementary materials for details on the measures). Participants then completed both the online social evaluative threat and control conditions of the learning task (counterbalanced presentation order across participants), mood was assessed again following completion of the learning task in each condition. In the threat condition, mood was additionally measured after receiving audio recording instructions and after completing the audio recording and ratings. After completing the learning task in the threat condition participants were asked: “Did you believe the ratings were real?” and “Were you worried about the ratings?”. Participants responded on a visual analogue scale ranging from 0 (*I was 100% sure they were NOT real* and *I was not worried at all* respectively) to 100 (*I was 100% sure they were real* and *I was very worried* respectively). Participant responses to the two manipulation check questions for the online social evaluative threat paradigm indicated average ratings of believability (*M* = 54.07, *SD* = 29.49) and worry (*M* = 50.67, *SD* = 30.01). Controlling the best fitting models for believability did not alter the pattern of results (see supplementary materials, Tables S3–S9) nor did excluding participants with manipulation check scores below 50 (out of 100). All participants were therefore retained for analysis. Participants were then presented with the debrief statement, which explained the study’s manipulations (i.e., their audio recording could not be accessed by other participants; the audio they rated was pre-recorded by a confederate; the views/comments tracker was not real). After the debrief, participants were asked to provide informed consent once again.

### Online social evaluative threat task

In the novel online social evaluation task participants completed a learning task twice, once in the threat and once in the control condition. The conditions’ presentation order was counter-balanced across participants. In both conditions of the learning task, participants were first shown a target shape for 1000 ms, followed by a fixation cross displayed for 500 ms. Participants were then presented with an array of two or four abstract shapes and were required to determine whether the target shape was present or absent in the array by clicking a corresponding button on the bottom of the screen (self-paced).

#### Threat condition

In the threat condition participants were instructed to complete a one-minute voice recording introducing themselves and their goals for the future prior to commencing the learning task. They were informed that their recording would be uploaded to the study’s database, and that it may be rated by other participants on three dimensions: attractiveness, intelligence, and friendliness. Recordings were only collected to track satisfactory completion of the task and were never made available to other participants. After being asked to spend one minute preparing what they would like to say during their recording, participants were instructed to test their microphones by completing a 4-s audio recording that they could play back and re-record if needed. They were then prompted to complete the recording that would be uploaded to the study’s database. To increase the authenticity of the threat, after completing their own recording participants were asked to select and rate a recording from the available database completed by other participants. For further details see supplementary materials.

After completing the recording component of the threat condition, participants completed the learning task and saw a “views and ratings tracker” on the bottom of their screen across all trials. Participants were informed that this tracker indicated the number of other participants who were currently listening to and rating recordings. The tracker began at 0 views and 0 ratings, and participants were informed that if these numbers remained at 0, it indicated that no other participants were currently online and completing the experiment. The tracker was in fact set to change on random trials to mimic a digital tracker with increasing views and ratings. Following the learning task, participants were informed that their recordings would be removed from the database and would no longer be available for listening or rating by other participants. Thereby, eliminating the evaluative threat.

#### Control condition

In the control condition, participants completed the learning task and saw a display tracking their current trial number on the bottom of their screen. The display was perceptually matched the comment tracker in the threat condition.

## Measures

### Mood

Mood was assessed as self-reported levels of stress, anxiety, and pleasantness on a visual analogue scale ranging from 0 (*not stressed/anxious/pleasant at all*) to 100 (*extremely stressed/anxious/pleasant*). Mood was measured at five timepoints throughout the study (see Figure S1 for flow chart of study procedure): at the start of the study (baseline), following the threat instructions, following the recording, following the task under threat, following the task in the control condition.

### Social rejection sensitivity

The Online and Offline Social Sensitivity Scale (O^2^S^3^)^24^ was used to assess self-reported social rejection sensitivity. The 18-item scale assesses social rejection sensitivity in both offline and online contexts (example item: “I worry about what others think of me”). Items are rated on a 4-point Likert scale ranging from 0 (*strongly disagree*) to 3 (*strongly agree*). The scale showed good internal consistency (ω_T_ = 0.90). For further details see SOM.

### Mental health

The primary mental health outcome of interest was depressive symptoms measured with the 7-item depression subscale of the short Depression Anxiety Stress Scales (DASS-21)^26^. The subscale demonstrated good internal consistency (ω_T_ = 0.93) in the current sample. As per protocol, the analyses were repeated including a composite mental health measure including the DASS-21 total score as well as the short Warwick-Edinburgh Mental Wellbeing Scale (WEMWS)^27^ and Strengths and Difficulties Questionnaire (SDQ)^28^. For details on these scales see the supplementary materials. PROCEDURES See Figure S1 for a schematic overview of the procedures. Following informed consent participants completed a baseline measure of mood, followed by self-report measures of demographics, mental health, social rejection sensitivity, and perceived social support. Next, participants completed the Raven’s Advanced Progressive Matrices and the affective digit span task (see supplementary materials for details on the measures). Participants then completed both the online social evaluative threat and control conditions of the learning task (counterbalanced presentation order across participants), mood was assessed again following completion of the learning task in each condition. In the threat condition, mood was additionally measured after receiving audio recording instructions and after completing the audio recording and ratings. After completing the learning task in the threat condition participants were asked: “Did you believe the ratings were real?” and “Were you worried about the ratings?”. Participants responded on a visual analogue scale ranging from 0 (I was 100% sure they were NOT real and I was not worried at all respectively) to 100 (I was 100% sure they were real and I was very worried respectively). Participant responses to the two manipulation check questions for the online social evaluative threat paradigm indicated average ratings of believability (M = 54.07, SD = 29.49) and worry (M = 50.67, SD = 30.01). Controlling the best fitting models for believability did not alter the pattern of results (see supplementary materials, Tables S3–S9) nor did excluding participants with manipulation check scores below 50 (out of 100). All participants were therefore retained for analysis. Participants were then presented with the debrief statement, which explained the study’s manipulations (i.e., their audio recording could not be accessed by other participants; the audio they rated was pre-recorded by a confederate; the views/comments tracker was not real). After the debrief, participants were asked to provide informed consent once again.

### Analytic strategy

All statistical analyses were conducted using R version 4.0.1 (packages noted in supplementary materials). Unless otherwise specified, all reported statistics were based on linear mixed models, including participant ID as random effect. In models including Mood as the outcome variable, *Condition* was modelled as baseline, post-threat (mood following completion of the learning task in the threat condition) and post-control (mood following completion of the learning task in the control condition). In models including Learning as the outcome, *Condition* was modelled as threat = 0 vs. control = 1 and *Time* was modelled as timepoints 1–4 in the learning task. Model fits were compared, a more complex model was chosen only if the model comparison was significant, and the Akaike Information Criterion was 2 points (or more) lower than the simpler model^29^. The third hypothesis was tested using mediation analysis. It should be noted here that mediation was used as a statistical analysis tool, to avoid artificially partitioning social rejection sensitivity into categories. It does not, however, imply causal or temporal mediation as the study is cross-sectional. To improve the inferences of non-significant results, Bayes factors were calculated using the Bayesian Information Criterion (BIC)^30^. All analyses including depressive symptoms were repeated including mental health (composite score) as a fixed effect, as per the pre-registration. These analyses showed the same pattern of results and are therefore included in the supplementary materials (Tables S8/S9).

Additionally, as per pre-registration, linear mixed models were run to investigate the effects of age on mood and learning. Moreover, we investigated whether any differences in learning rates could simply be accounted for by age-related variance in cognitive ability^31^ or affective control (the capacity to deploy cognitive control in affective contexts)^32^. To do so, set I of the Raven’s Advanced Progressive Matrices^31^ was used to assess cognitive abilities. The affective backward digit span task^33^ was used to measure affective control. There were no significant associations between affective control and mood or learning (Table S2), but cognitive ability was significantly associated with learning in the threat condition (*r* =  − 0.15, *p* = 0.018), such that participants with higher cognitive ability demonstrated a decreased slope (indicating increased learning) in the threat condition of the learning task. Cognitive ability was therefore entered as a covariate in analyses including learning.

## Results

### Effect of online social evaluative threat on mood

**Figure 1:**
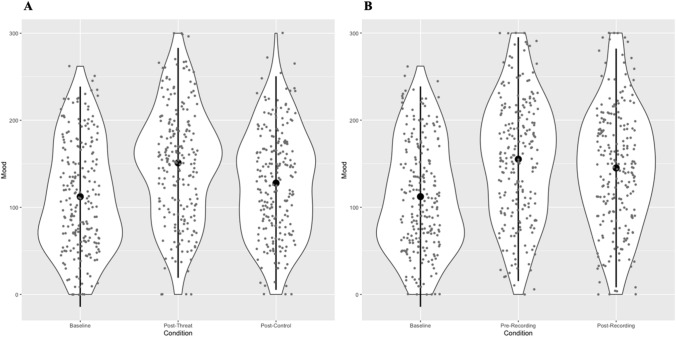
Effects of online social evaluative threat on mood. *Note* The figure shows changes in participants’ self-reported mood (with mean and SD) at different time points throughout the study. Mood is operationalised as the sum of anxiety (out of 100), stress (out of 100), and reverse-coded pleasantness (out of 100) measures, with higher scores indicating greater negative mood. In (**A**) condition is modelled as baseline, post-threat (mood following completion of the learning task threat condition) and post-control (mood following completion of the learning task control condition). In (**B**) condition is modelled as baseline, pre-recording (immediately prior to recording self-introduction audio) and post-recording (immediately following recording of self-introduction audio and rating of peer self-introduction audio). Table S1 includes all condition means.

As shown in Fig. 1A, a linear mixed model investigating the effect of condition (baseline, post-threat, post-control) on mood, showed that participants reported heightened negative mood following the threat condition compared to baseline, *b* = 38.86, *SE* = 3.74, *t* = 10.38, *p* < 0.001. As shown in Fig. 1B, participants also reported heightened negative mood following the control condition compared to baseline, *b* = 15.65, *SE* = 3.74, *t* = 4.18, *p* < 0.001. Comparing the two task conditions showed, in line with the task design, that participants reported significantly more negative mood following the threat condition compared to the control condition, *b* = 23.20, *SE* = 3.75, *t* = 6.19, *p* < 0.001.

When exploring mood within the evaluative threat condition, results showed a significant increase in reported negative mood from baseline to post-instruction (immediately prior to recording self-introduction audio; *b* = 42.98, *SE* = 3.74, *t* = 11.50, *p* < 0.001), and from baseline to post-recording (immediately following recording of self-introduction audio and rating of peer self-introduction audio; *b* = 32.89, *SE* = 3.74, *t* = 8.80, *p* < 0.001). Comparing pre-recording mood to post-recording mood showed that participants reported a significant decrease in negative mood following recording completion, *b* = -10.10, *SE* = 3.75, *t* = 2.69, *p* = 0.022.

### Effects of social risk (social rejection sensitivity) and protective (social support) factors under online social evaluative threat

#### Effects on mood

**Figure 2:**
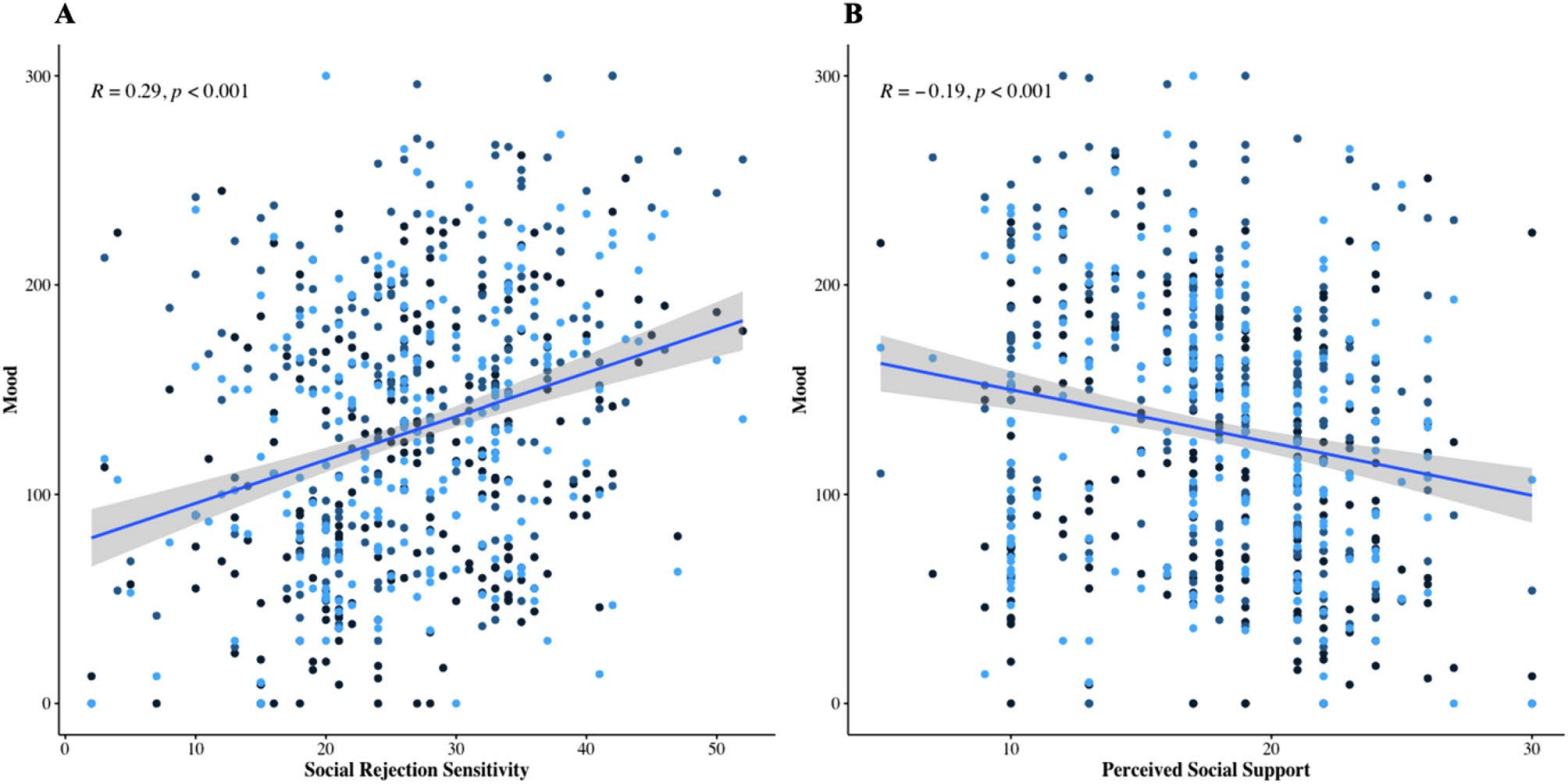
Effects of social rejection sensitivity and perceived social support on mood.* Note* Mood is operationalised as the sum of anxiety (out of 100), stress (out of 100), and reverse-coded pleasantness (out of 100) measures, with higher scores indicating greater negative mood. (**A**) illustrates the association between participant mood and level of social rejection sensitivity (total score on the Online and Offline Social Sensitivity Scale)^24^ after controlling for condition. (**B**) illustrates the association between participant mood (controlling for condition) and level of perceived social support (combined score of the family and friends subscales from the Schuster Social Support Scale)^34^.

When investigating the effects of social risk and protective factors on mood, the most parsimonious fit was the model including condition (baseline, post-threat, post-control), social rejection sensitivity (Table 2, Model 2A) and social support (Table 2, Model 2B), respectively, but no interaction terms. As shown in Fig. 2A and B respectively, significant main effects of social rejection sensitivity and social support on mood (*BFs* > 100) showed that on average, participants’ reported negative mood increased as a function of increased social rejection sensitivity and decreased as a function of increased perceived social support.

**Table 2:** Models investigating the effects of condition, social rejection sensitivity (models 2/3A) and social support (Models 2/3B) on mood.

Dependent variable	Predictor variable(s)	*b*	df	*t*	*p*	*R*^*2*^*m/R*^*2*^*c*	*AIC*	*Chi*^*2*^	*P*_*(chi2)*_
**Model 1**									
						0.01/0.56	7258.5		
Mood	Condition	7.83	447.43	3.81	< 0.001***				
**Model 2A**									
						0.09/0.56	7233.1	27.42	< .001***
Mood	Condition	7.83	447.55	3.81	< 0.001***				
	Social rejection sensitivity	2.04	221.38	5.40	< 0.001***				
**Model 3A**									
						0.09/0.56	7235.0	0.11	.738
Mood	Condition	5.81	447.55	0.91	0.363				
	Social rejection sensitivity	1.89	647.09	3.21	0.001**				
	Condition × Social rejection sensitivity	0.08	447.55	0.33	0.738				
**Model 2B**									
						0.08/0.56	7237.6	22.94	< .001***
Mood	Condition	7.83	448.59	3.81	< 0.001***				
	Social support	− 3.99	222.57	− 4.91	< 0.001***				
**Model 3B**									
						0.08/0.56	7239.6	0.00	.959
Mood	Condition	7.55	448.59	1.30	0.195				
	Social support	− 3.94	644.85	− 3.13	0.002**				
	Condition × Social support	− 0.02	448.59	− 0.05	0.959				

*p < 0.05 **p < 0.01 ***p < 0.001. Mood is operationalised as the sum of anxiety (out of 100), stress (out of 100), and reverse-coded pleasantness (out of 100) measures, with higher scores indicating greater negative mood. Condition is modelled as baseline, post-threat (mood following completion of the learning task threat condition) and post-control (mood following completion of the learning task control condition). Models 2A and 3A included social rejection sensitivity as predictor, and models 2B and 3B included social support as predictor.

#### Effects on learning

The best fitting model for learning was the model including only time (Table 3, Model 1). However, there was no significant main effect of time on learning (*BF* = 0.035). This suggests that learning did not take place in the present study. As per the study’s pre-registration, all further analyses investigating the effects of social rejection sensitivity, social support, depressive symptoms, and mental health symptoms on learning were run. However, due to the absence of learning these analyses have been reported in the supplementary materials (Tables S4/S9).

**Table 3:** Models investigating the effects of time and condition on learning.

Dependent Variable	Predictor Variable(s)	*b*	df	*t*	*p*	*R*^*2*^*m/R*^*2*^*c*	*AIC*	*Chi*^*2*^	*p*_*(chi2)*_
**Model 1**									
						0.00/0.01	32,307.3		
Slope	Time	15.05	1791.84	0.95	0.344				
	Cognitive ability	− 20.39	304.99	− 2.63	0.009**				
**Model 2**									
						0.00/0.01	32,309.1	0.14	.713
Slope	Time	15.27	1794.38	0.96	0.337				
	Condition	− 13.70	1792.90	− 0.39	0.699				
	Cognitive ability	− 19.95	306.47	− 2.58	0.010*				
**Model 3**									
						0.01/0.01	32,304.2	1.82	.177
Slope	Time	− 9.64	1798.52	− 0.43	0.670				
	Condition	− 136.08	1791.19	− 1.57	0.1167				
	Cognitive ability	− 19.83	306.38	− 2.56	0.011*				
	Time x Condition	49.09	1793.04	1.55	0.123				

*p < 0.05 **p < 0.01 ***p < 0.001. Slope indicates average learning on learning task trials. Time indicates time points 1 through 4 in the learning task. Condition indicates condition of the learning task (coded 0 = threat, 1 = control). Cognitive ability was measured with the Raven’s Advanced Progressive Matrices^31^.

Given the absence of learning, exploratory analyses were run to investigate the effects of social rejection sensitivity and social support on overall task performance (reaction time and accuracy), as an index of cognitive functioning with a Bonferroni-corrected significance level of *p* < 0.025 applied for the two additional outcomes.

For reaction time the baseline models with time showed the most parsimonious fit for both models including social sensitivity and social support (Model 1; Table 4). There was a significant main effect of time on reaction time (*BF* = 41.42), such that participants became faster over the course of the task.

**Table 4:** Model Fit Analyses for Reaction Time and Accuracy.

	*AIC*	*R*^*2*^*m/R*^*2*^*c*	*Chi*^*2*^	*p*_*(chi2)*_
**Reaction time**				
Model 1	32,244.1	0.01/0.20		
Model 2	32,241.6	0.01/0.20	2.80	0.094
Model 3A	32,241.4	0.01/0.20	6.71	0.035
Model 4A	32,245.1	0.01/0.20	2.60	0.626
Model 3B	32,243.0	0.01/0.20	3.41	0.182
Model 4B	32,245.3	0.01/0.20	9.14	0.166
**Accuracy**				
Model 1	8057.0	0.02/0.56		
Model 2	8048.6	0.02/0.56	11.00	< 0.001***
Model 3A	8049.3	0.02/0.56	0.69	0.408
Model 4A	8049.3	0.03/0.56	8.70	0.122
Model 3B	8041.2	0.04/0.56	9.56	0.002**
Model 4B	8041.8	0.04/0.56	6.62	0.157

*p < 0.025 ** *p* < .01 *** *p* < 0.001. A full summary of regression models of reaction time exploratory analyses have been reported in the supplementary materials (Tables S5/S6).

For accuracy the best fit was for a model including time and condition (Model 2, Table 4), when investigating social rejection sensitivity. A significant main effect of time (*BF* > 100) showed increasing accuracy over time. The significant main effect of condition on accuracy (*BF* = 5.482) showed that participants were more accurate in the control compared to the threat condition. When investigating the effects of social support on accuracy, the best fit was the model including time, condition, and social support, but no interactions (Model 3B, Table 4). In this model, the effects of time and condition on accuracy remained significant. Additionally, after controlling for time and condition, there was a significant main effect of social support on accuracy (*BF* = 2.67), such that increased perceived social support was associated with greater accuracy on the learning task.

#### Effects of pre-existing depressive symptoms under online social evaluative threat

When investigating the effects of depressive symptoms on mood during the experiment, the most parsimonious fit was the model including only condition (baseline minus post-threat vs baseline minus post-control), but not including depressive symptoms or interactions between the two (Table S7, Model 1). This indicates that, contrary to our hypotheses, the effect of condition on mood in the task did not significantly differ as a function of pre-existing depressive symptoms. Given the absence of an effect of pre-existing depressive symptoms hypothesis 3 was no longer warranted. However, as it is pre-registered we report the results in the supplementary analyses.

#### Effects of age

Exploratory age analyses indicated that there was no significant effect of age on mood (*b* = − 0.70, *SE* = 1.28, *t* = − 0.55, *p* = 0.586) or significant interaction between age and condition (mood: *b* = 0.05, *SE* = 0.48, *t* = 0.11, *p* = 0. 914, *BF* = 0.039). Exploratory analyses indicated that there was also no significant effect of age on accuracy (*b* = 0.14, *SE* = 0.12, *t* = 1.14, *p* = 0.255) or reaction time (*b* = − 8.96, *SE* = 23.05, *t* = − 0.39, *p* = 0.698), or significant interaction between age and condition (accuracy: *b* = − 0.04, *SE* = 0.09, *t* = − 0.53, *p* = 0.597, *BF* = 0.055; reaction time: *b* = − 45.71, *SE* = 30.89, *t* = − 1.48, *p* = 0.140, *BF* = 0.141).

## Discussion

This pre-registered study tested the effects of online social evaluative threat on adolescent mood and learning. In line with our predictions, participants reported increased negative mood following an online social evaluative threat. Mood also varied as a function of social risk and protective factors. That is, negative mood was highest in individuals high on social rejection sensitivity and lowest in those high on perceived social support. In offline social contexts, social evaluative threat during public speaking tasks has been associated with psychological (e.g., negative affect) and physiological (e.g., elevated cortisol) correlates of distress^35–37^. The present study suggests that online social evaluation may be perceived as similarly stressful.

Although we did observe an overall effect of social evaluative threat, this did not vary as a function of hypothesised factors: risk (social rejection sensitivity) and resilience (social support), nor did it vary as a function of age. The lack of an effect of age was surprising, given that previous research reports that younger age has been associated with greater self-reported distress on social stress tasks^36^. Adolescents have been shown to be more sensitive to social stressors^38^. Our finding of no differential effects for different age groups may suggest that online social evaluation is a potent stressor across ages.

The effect of social evaluative threat on mood also did not vary by individuals’ social rejection sensitivity or social support (*Hypothesis 1a*). That is, individuals who described themselves as more or less sensitive to social rejection or more or less socially supported did not react differently to social evaluative threat. In line with this finding, previous research showed that these social factors were associated with mood across contexts, supporting their role in affect more generally^13,39^. The present findings suggest that social evaluative threat might be experienced as stressful regardless of pre-existing social risk and resilience factors.

While the effects of social evaluative threat on learning (*Hypothesis 1b*) could not be examined as set out in our pre-registration, as learning did not occur on the task, we did explore effects on cognitive functioning (i.e., accuracy and reaction time in the learning task). Bearing the caveat in mind that these analyses were exploratory and thus need replication, the finding that accuracy on the learning task was significantly reduced in the context of online social evaluative threat suggests that online social evaluation may disrupt cognitive functioning. This finding has potential implications across domains of education, development and cognitive sciences. In line with attentional control theory^16^, the perpetual potential for online social evaluation creates an environment in which cognitive resources are partially deployed to attend to potential social evaluation and to the affective response elicited by this social threat, thereby limiting resources to acquire academic and vocational skills that depend on good cognitive functioning. Furthermore, we observed that this effect did not vary across adolescence and young adulthood, which are primary developmental periods for academic and vocational skill development^40^.

The lack of observed effects of depressive symptoms (*Hypothesis 2*) was surprising, especially as previous studies have found that depressive symptoms are related to increased feelings of stress and anxiety^41–43^, both of which were included as factors in this study’s mood variable and have been shown to increase following social evaluative threats^37^. However, we found that social rejection sensitivity partially accounted for a relationship between depressive symptoms and mood (*Hypothesis 3*), in line with studies reporting associations between social rejection sensitivity, increased depressive symptoms and decreased well-being^44^. It is important to note that as no direct effects of depressive symptoms on participant mood were observed, significant results from mediation analyses investigating the effects of other factors on this relationship are preliminary, with future studies needing to further investigate these effects through longitudinal experimental designs.

## Limitations and directions for future research

While the present study provides valuable insights into the relationship between social evaluative threat and adolescent mood in an online context, as well as providing preliminary evidence of an effect of online social evaluative threat on cognition, its limitations must be considered when interpreting the results. Firstly, we are unable to make any causal or directional inferences from the study’s findings regarding the mediating analyses investigating the relationship between social rejection sensitivity, mental health, and mood, as the study was conducted cross-sectionally and the predictor (mental health) and mediator (social rejection sensitivity) were concurrently measured. Furthermore, the absence of learning on the task suggests that more trials are needed to detect learning and future research is needed to investigate the effect of evaluative threat on learning specifically.

In sum, this pre-registered study suggests that online social evaluative threat impacts mood and cognition adversely. These insights contribute to an evidence base to inform decision-making by young people, parents, educators and policymakers regarding social media use.

## Supplementary Information

Supplementary Information.

## Data Availability

The dataset generated and analysed during the current study available from the corresponding author on reasonable request.
