# Supplementary material for: The effect of online social evaluation on mood and cognition in young people
Source: Sci Rep. 2022 Dec 5;12:20999. doi: 10.1038/s41598-022-24932-w (PMC9723113; doi:10.1038/s41598-022-24932-w)
Supplement: Supplementary file 1 — Supplementary Information. [file 41598_2022_24932_MOESM1_ESM.docx]

The Effect of Online Social Evaluation on Mood and Cognition in Young People

Supplementary Materials

Excluded Participants

93 participants were excluded from the study due to one or more of the following reasons. Participants who reported previous traumatic brain injury or a diagnosed learning disability were automatically excluded prior to commencing the study; one participant reported a lack of English fluency; 11 participants were above 30 years of age; 32 participants answered more than 1 attention check item incorrectly; 46 participants completed the study multiple times; 62 participants performed below chance on the learning task (i.e., they scored 40 or less responses correctly out of the 80 trials); and 30 participants performed at a mean response time in the learning task above 20000ms or below 200ms (i.e. performance outliers)^1^.

Social Rejection Sensitivity Measure

The Interpersonal Sensitivity Measure^27^ was additionally administered to validate the O^2^S^3^ in the current study, as the scale had not been published when this project was pre-registered. It is a 36-item scale measuring hypersensitivity to interpersonal rejection through 5 subscales: interpersonal awareness, need for approval, separation anxiety, timidity, and fragile inner self. Participants indicate the extent to which each item applies to them on a 4-point Likert scale ranging from 0 (very unlike) to 3 (very like). In the present study, the measure showed good internal consistency (ωT = .84) and supported the construct validity of the O^2^S^3^ (r = .68).

Social Support

The Schuster Social Support Scale^28^ was used to assess perceived social support. The friend and family subscales were included in the present study, as studies have indicated that parents and friends are among the sources of support that have the strongest association with adolescents’ well-being^29^. The subscales consist of 5 parallel items for both friends and family, which measure supportive interactions (e.g. “How often do friends/family make you feel cared for?”) and negative interactions (e.g. “How often do friends/family criticize you?”). Participants indicate how often they experience each item on a 4-point Likert scale ranging from 1 (*often*) to 4 (*never*). The scale demonstrated good internal consistency (ω_T_ = .83) in the present sample.

Mental Health Measures

The short Warwick-Edinburgh Mental Wellbeing Scale (WEMWS)^2^ was used to measure emotional wellbeing. The 7-item scale is scored on a 5-point Likert scale ranging from 1 (*none of the time*) to 5 (*all of the time*). In the present study, the scale demonstrated good internal consistency (ω_T_ = .88).

The Strengths and Difficulties Questionnaire (SDQ)^3^ was used to assess symptoms of internalizing and externalizing disorders. The 25-item questionnaire is divided into five subscales, four of which measure difficulties (emotional symptoms, conduct problems, hyperactivity/inattention, peer relationship problems) and one of which measures strengths (prosocial behaviour). Participants indicate how much each statement applied to themselves on a 3-point Likert scale ranging from 0 (*not true*) to 2 (*certainly true*). The Difficulties score (combined total score on the emotional symptoms, conduct problems, and hyperactivity/inattention subscales) was used in the present study, as it has been shown to be strongly associated with estimates of mental health disorders^4^. The questionnaire showed acceptable internal consistency (ω_T_ = .77) in the current sample.

Emotional disorder symptomatology was additionally assessed using the short Depression Anxiety Stress Scales (DASS-21)^5^. The DASS-21 is made up of three 7-item subscales that measure symptoms of depression, anxiety and stress. Items are rated on a 4-point Likert scale ranging from 0 (*did not apply to me at all*) to 3 (*applied to me very much, or most of the time*). In the present study, the DASS-21 showed good internal consistency (ω_T_ = .95).

The three measures’ total scores were standardised and then combined to generate a composite mental health score. A higher mental health score indicated more mental health problems.

Details and Modifications of the Learning Task

This task utilised consistent mapping, where the target shapes were always targets, and distractor shapes were always distractors. Targets were included in half of the trials. Condition presentation order of the two learning task conditions was determined by system-generated random assignment using the web application available at <https://gorilla.sc/>, with half of the participants completing the control condition first, and the other half completing the threat condition first. Additionally, two versions (A and B) of the learning task were created with the same targets and distractors presented in different combinations and orders. This was done to avoid training effects as participants completed each of the two conditions. Participants completed one version (e.g., A) during their first completion of the learning task, and another version (e.g., B) during their second completion of the learning task. Block randomisation was used such that participants were randomly assigned to one of the four equally sized, predetermined blocks. General linear model (continuous variables) and non-parametric (dichotomous variables) analyses showed successful randomisation with no significant differences in demographics, clinical and social characteristics across the different pathways: age, *F*(3,251) = .06, *p* = .980, gender, ***χ*** *^2^*(9, *N* = 255) = 11.73, *p* = .229), ethnicity, ***χ*** *^2^*(21, *N* = 255) = 17.56, *p* = .677, level of education, ***χ****^2^*(9, *N* = 255) = 5.92, *p* = .748, depressive symptoms, *F*(3,251) = .339, *p* = .797, mental health, *F*(3,251) = .25, *p* = .864, SRS, *F*(3,251) = .34, *p* = .796, and perceived social support, *F*(3,251) = 0.436, *p* = .727.

*Modifications.* While the original task was made up of 6-8 learning blocks of 80 trials, the present task was modified to a single learning block of 80 trials. This modification allowed the total study length to remain under one hour, making it less likely that participants may become fatigued or disinterested and discontinue the experiment. Additionally, while the original task used Chinese characters printed in black ink on a white background as the task stimuli, the present task utilised coloured shapes on a white background sourced from the matrix reasoning item bank^6^. As there is a large Chinese-speaking community in Australia, with Mandarin alone reported as the second most commonly spoken language at home^7^, using the original stimuli may have affected the results, as participants who were familiar with the stimuli might then distinguish between target and distractor stimuli more easily, thus aiding task performance.

Details Social Evaluative Threat Condition

All available recordings were in fact pre-recorded by confederates and based on profiles of fictional high school and university students. These recordings were matched to participants’ age group, such that participants aged 17 and below chose from recordings of high school students, whereas participants aged 18 and above chose from recordings of university students. After selecting and listening to this recording, participants were asked to rate how attractive, intelligent, and friendly the other participant sounded on visual analogue scales ranging from 0 to 100.

Measures of Cognitive Ability and Affective Control

***Cognitive Functioning.*** Set I of the Raven’s Advanced Progressive Matrices^8^ was used to assess cognitive abilities. The task presents an incomplete pattern matrix. Participants were required to select the design that completed the pattern of a 3x3 matrix of geometric designs from a set of 8 potential designs. The patterns increased in difficulty across 12 trials. In the current study, completion of the task was paced. 20 seconds for trials 1-8 and 40 seconds for trials 9-12, after which the task automatically moved to the next trial.

***Affective Control.*** The affective backward digit span task^9^ was used to measure affective control. This was a modified version of the standard backward digit span task^10^, where participants are required to repeat sequentially presented digits in reverse order. In the present study, the digits were superimposed over mildly negative affective images (e.g. spiders), or neutral images (e.g. stairs) from the Geneva Affective Picture Database^11^. The digits were presented for 1500ms each, with a numerical keypad presented at the end of each trial for participants to enter the digits in reverse order^9^. Each span level was presented twice, with the task beginning with two digits per trial. Participants were required to answer at least one trial per span level correctly to proceed to the next span level. The task was terminated if both trials on a span level were incorrect. Affective control was operationalised as the relative number of digits recalled in the affective relative to the neutral condition, controlling for age-related variability in general task performance by dividing the difference score (calculated by subtracting the neutral score from the affective score) by the neutral score.

**Figure S1**

*Study procedure flow chart.*

*
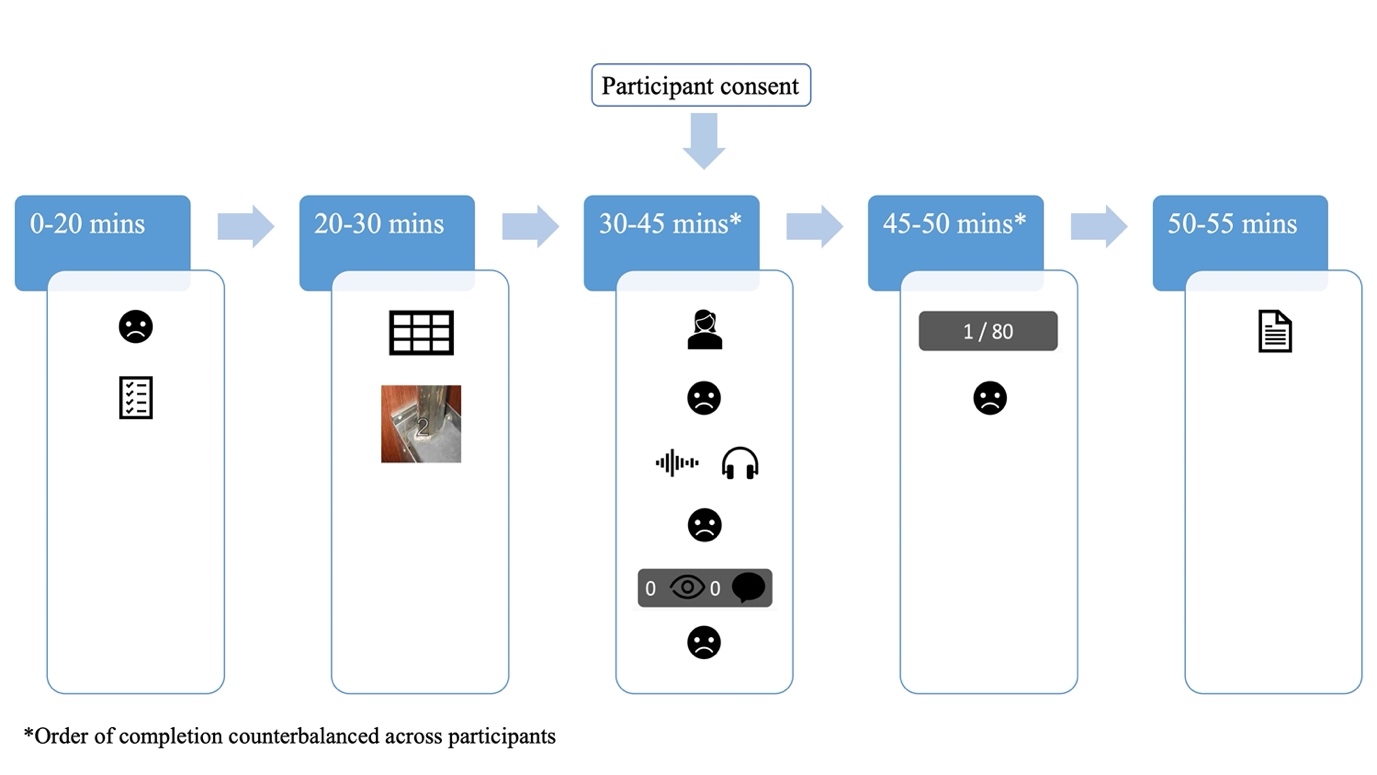
*

*Note:* The figure depicts the study procedure.
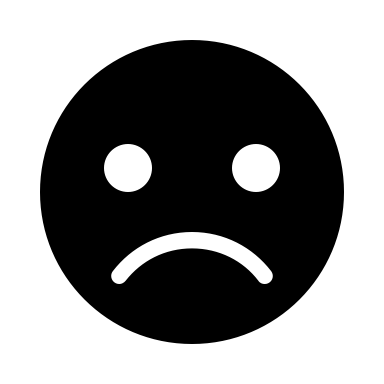
 = mood measure (sum of anxiety, stress, and reverse-coded pleasantness measures);
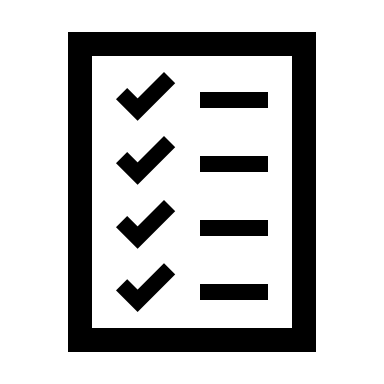
 = self-report measures (demographics questionnaire, DASS-21, WEMWS, SDQ, O^2^S^3^, ISS and Schuster Social Support Scale);
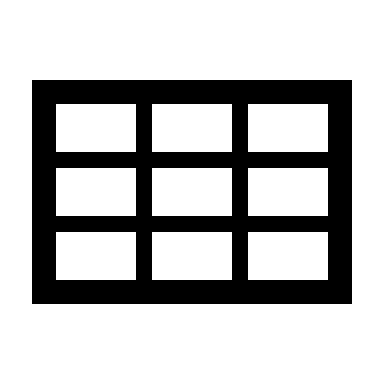
 = Raven’s Advanced Progressive Matrices Set I;
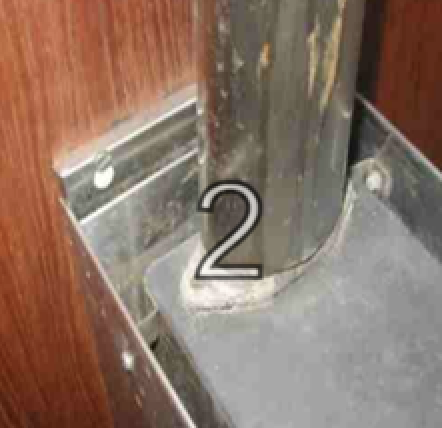
 = affective backward digit span task;
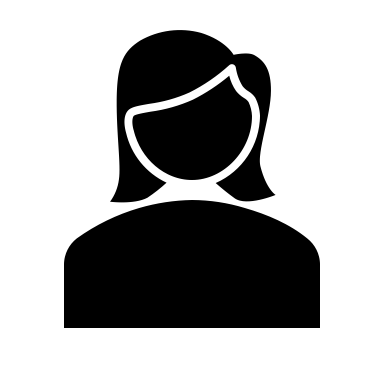
 = instructions for social evaluative threat activity;
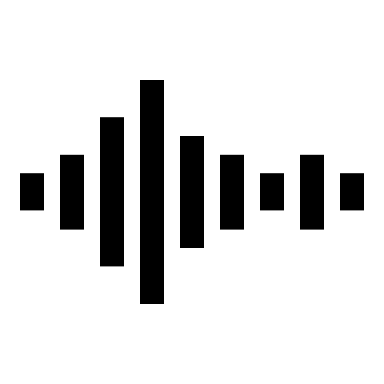
 = self-introduction audio recording;
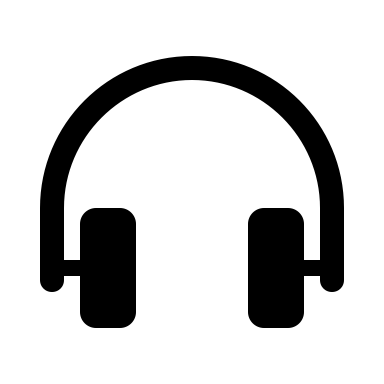
 = listening and rating others’ self-introduction;
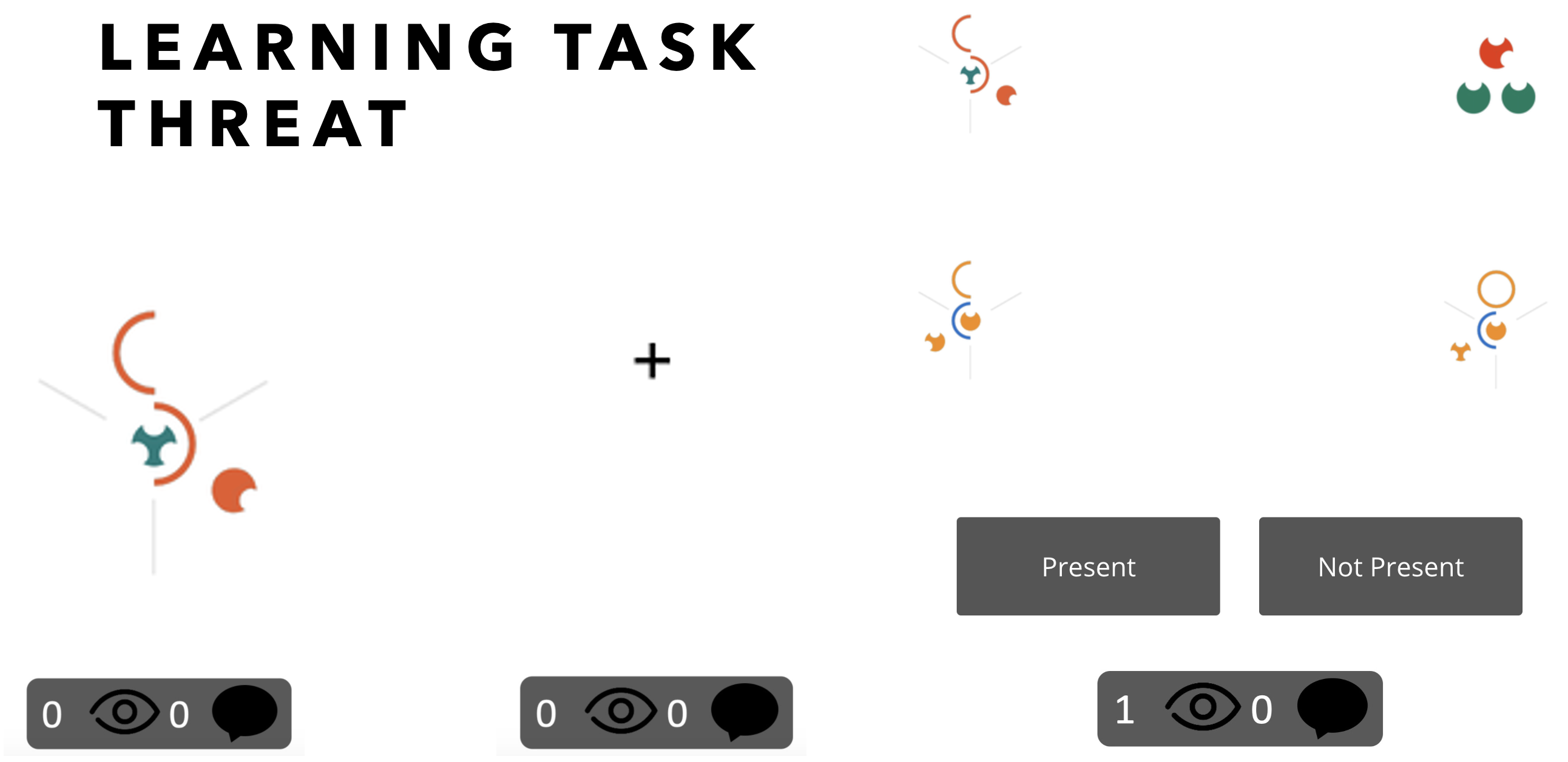
 = threat learning task condition;
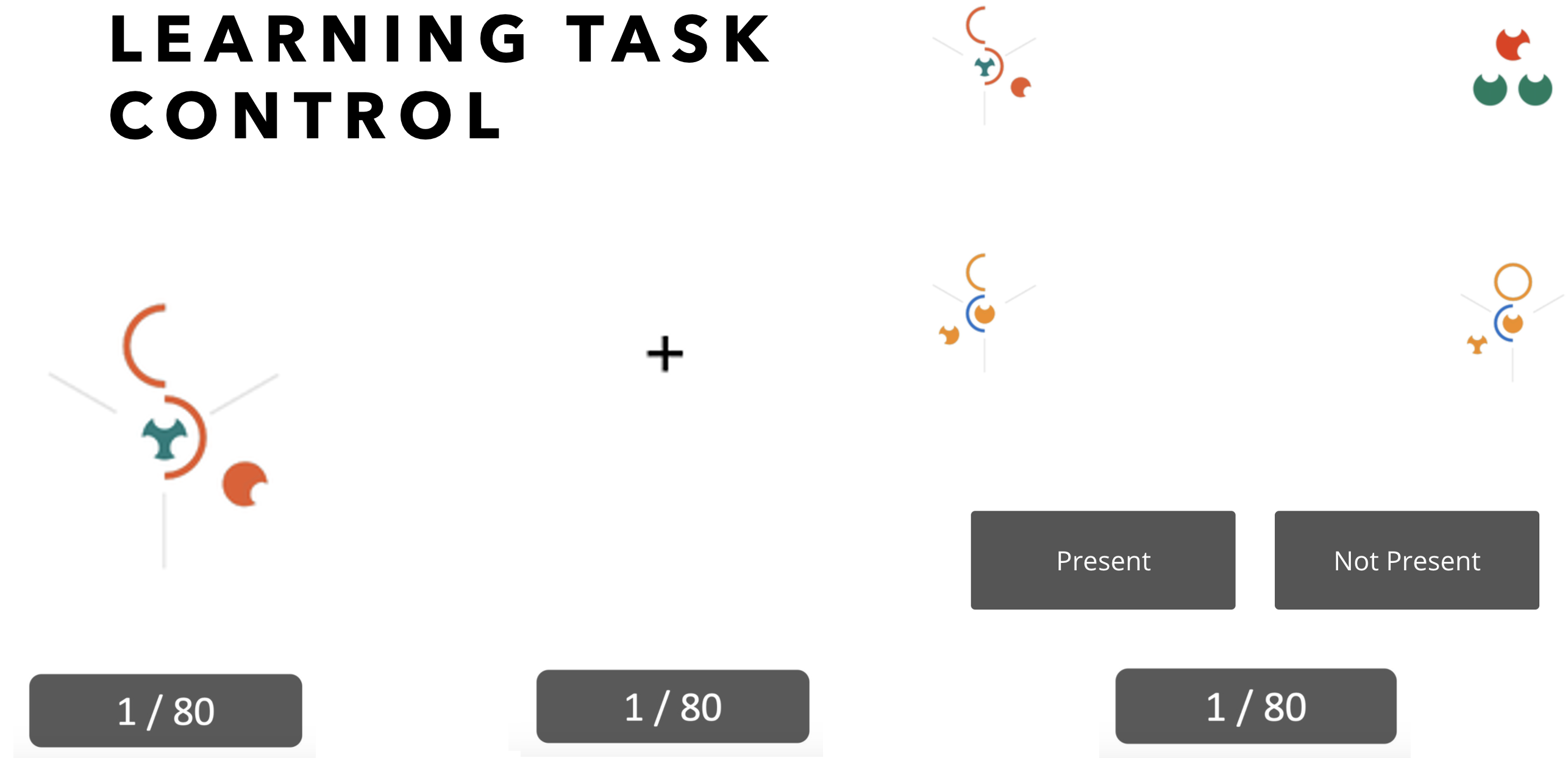
 = control learning task condition;
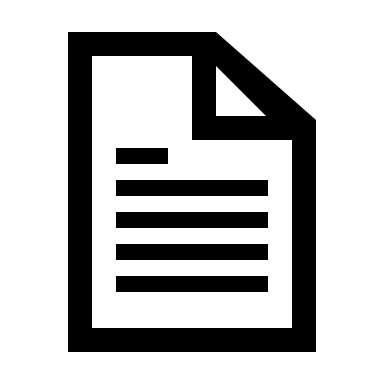
 = debrief.

R-packages

Analyses were conducted including the following packages. Linear mixed models were conducted using the lme4 R package^12^. Any significant effects were further investigated with planned contrasts using the lsmeans package in R^13^. Model comparisons were conducted using the dplyr R package^14^. R-squared values were also calculated for each model using the rsq package^15^. The third hypothesis was tested using mediation analyses run with the mediation^16^ and lavaan^17^ packages.

Supplementary Results

**Table S1**

*Means and standard deviations of variables*

| Variable | *M* (*SD*) |
| --- | --- |
| SRS | 26.75 (9.16) |
| Support | 18.40 (4.34) |
| Depressive Symptoms | 14.96 (9.81) |
| Health | -0.25 (2.72) |
| Raven’s | 7.60 (2.34) |
| Digit Span | 0.07 (0.51) |
| Mood Measures |  |
| Baseline | 115.99 (64.11) |
| Pre-Recording | 157.47 (69.65) |
| Post-Recording | 148.47 (69.54) |
| Post-Threat | 153.54 (67.29) |
| Post-Control | 130.84 (61.94) |
| Learning Task Measures |  |
| Threat Slope | 161.70 (457.85) |
| Control Slope | 153.80 (381.88) |
| Threat RT | 3115.83 (2039.00) |
| Control RT | 1449.64 (525.79) |
| Threat Accuracy | 68.75 (8.64) |
| Control Accuracy | 69.90 (7.78) |

Note. SRS = social rejection sensitivity; Support = perceived social support; Health = composite mental health score (standardized and summed WEMWS, SDQ and DASS-21 scores); Raven’s = cognitive ability; Digit span = affective control; Mood Measures = sum of anxiety (out of 100), stress (out of 100), and reverse-coded pleasantness (out of 100) measures (higher scores indicate greater negative mood); Pre-Recording = immediately prior to recording self-introduction audio; Post-Recording = immediately following recording of self-introduction audio and rating of peer self-introduction audio; Post-Threat = following completion of the learning task threat condition; Post-Control = following completion of the learning task control condition; Threat Slope = threat learning condition slope; Control Slope = control learning condition slope; Threat Accuracy = correct responses on threat learning condition; Control Accuracy = correct responses on control learning condition; Threat RT = reaction time on threat learning condition; Control RT = reaction time on control learning condition.

**Table S2**

*Correlations of predictor and outcome variables.*

| Variable | 1 | 2 | 3 | 4 | 5 | 6 | 7 | 8 | 9 | 10 |
| --- | --- | --- | --- | --- | --- | --- | --- | --- | --- | --- |
| 1. Age | - |  |  |  |  |  |  |  |  |  |
| 2. SRS | -.15* | - |  |  |  |  |  |  |  |  |
| 3. Support | .15* | -.26** | - |  |  |  |  |  |  |  |
| 4. Depressive Symptoms | -.06 | .44** | -.43** | - |  |  |  |  |  |  |
| 5. Raven’s | .11 | -.14* | .08 | -.22** | - |  |  |  |  |  |
| 6. Digit span | .03 | .01 | -.01 | .12 | .03 | - |  |  |  |  |
| 7. Post-Control Mood | -.01 | -.00 | -.01 | .08 | -.00 | .07 | - |  |  |  |
| 8. Post-Threat Mood | .09 | -.08 | .10 | -.02 | .03 | .07 | .64** | - |  |  |
| 9. Control Slope | -.02 | -.03 | -.09 | .01 | -.02 | .06 | -.09 | -.09 | - |  |
| 10. Threat Slope | .05 | .06 | -.02 | .13* | -.15* | -.05 | .04 | .07 | .04 | - |

Note. * p < .05, ** p < .01, *** p < .001; SRS = social rejection sensitivity; Support = perceived social support; Raven’s = cognitive ability; Digit span = affective control; Post-Control Mood = difference in mood from baseline to post-control; Post-Threat Mood = difference in mood from baseline to post-threat; Control Slope = average control learning condition slope; Threat Slope = average threat learning condition slope.

Best Fitting Models Including Believability Rating for Hypothesis 1 Analyses

**Table S3**

*Models Investigating the Effects of Condition, Social Rejection Sensitivity (Models 2/3A) and Social Support (Models 2/3B) on Mood*

|  | Predictor Variable(s) | *b* | df | *t* | *p* | *R^2^m/R^2^c* | *AIC* | *Chi^2^* | *P_(chi2)_* |
| --- | --- | --- | --- | --- | --- | --- | --- | --- | --- |
| **Model 1** | | | | | | | | | |
|  | | | | | | 0.01/0.56 | 7258.5 |  |  |
| Mood | Condition | 7.83 | 447.43 | 3.81 | <.001*** |  |  |  |  |
| **Model 2A** | | | | | | | | | |
|  | | | | | | 0.09/0.56 | 7235.1 | 27.44 | <.001*** |
| Mood | Condition | 7.83 | 447.57 | 3.81 | <.001*** |  |  |  |  |
|  | SRS | 2.04 | 222.40 | 5.41 | <.001*** |  |  |  |  |
|  | Believability | 0.02 | 221.61 | 0.17 | .864 |  |  |  |  |
| **Model 3A** | | | | | | | | | |
|  |  |  |  |  |  | 0.09/0.56 | 7235.0 | 0.11 | .738 |
| Mood | Condition | 5.81 | 447.55 | 0.91 | .363 |  |  |  |  |
|  | SRS | 1.89 | 647.09 | 3.21 | .001** |  |  |  |  |
|  | Condition x SRS | 0.08 | 447.55 | 0.33 | .738 |  |  |  |  |
| **Model 2B** | | | | | | | | | |
|  |  |  |  |  |  | 0.08/0.56 | 7239.6 | 22.97 | <.001*** |
| Mood | Condition | 7.83 | 448.57 | 3.81 | <.001*** |  |  |  |  |
|  | Support | -4.00 | 222.56 | -4.92 | <.001*** |  |  |  |  |
|  | Believability | -0.02 | 222.63 | -0.17 | .863 |  |  |  |  |
| **Model 3B** | | | | | | | | | |
|  |  |  |  |  |  | 0.08/0.56 | 7239.6 | 0.00 | .959 |
| Mood | Condition | 7.55 | 448.59 | 1.30 | .195 |  |  |  |  |
|  | Support | -3.94 | 644.85 | -3.13 | .002** |  |  |  |  |
|  | Condition x Support | -0.02 | 448.59 | -0.05 | .959 |  |  |  |  |

Note. * p < .05 ** p < .01 *** p < .001. Mood is operationalised as the sum of anxiety (out of 100), stress (out of 100), and reverse-coded pleasantness (out of 100) measures, with higher scores indicating greater negative mood. Condition is modelled as baseline, post-threat (mood following completion of the learning task threat condition) and post-control (mood following completion of the learning task control condition). Models 2A and 3A included social rejection sensitivity as predictor, and models 2B and 3B included social support as predictor. Believability indicates how believable participants found the study’s social evaluative threat.

**Table S4**

*Models Investigating the Effects of Time, Condition, Social Rejection Sensitivity (Models 3/4A) and Social Support (Models 3/4B) on Learning*

| Dependent Variable | Predictor Variable(s) | *b* | df | *t* | *p* | *R^2^m/R^2^c* | *AIC* | *Chi^2^* | *p_(chi2)_* |
| --- | --- | --- | --- | --- | --- | --- | --- | --- | --- |
| **Model 1** | | | | | | | | | |
|  |  |  |  |  |  | 0.00/ 0.01 | 32304.0 |  |  |
| Slope | Time | 15.29 | 1794.09 | 0.96 | .336 |  |  |  |  |
|  | Cognitive Ability | -20.39 | 304.99 | -2.63 | .009** |  |  |  |  |
|  | Believability | -0.52 | 295.02 | -0.85 | .398 |  |  |  |  |
| **Model 2** | | | | | | | | | |
|  |  |  |  |  |  | 0.00/ 0.01 | 32304.6 | 0.15 | .699 |
| Slope | Time | 15.27 | 1794.38 | 0.96 | .337 |  |  |  |  |
|  | Condition | -13.70 | 1792.90 | -0.39 | .699 |  |  |  |  |
|  | Cognitive Ability | -19.95 | 306.47 | -2.58 | .010* |  |  |  |  |
| **Model 3A** | | | | | | | | | |
|  |  |  |  |  |  | 0.00/ 0.01 | 32306.6 | 0.15 | .928 |
| Slope | Time | 15.27 | 1794.00 | 0.96 | .337 |  |  |  |  |
|  | Condition | -13.69 | 1792.63 | -0.39 | .699 |  |  |  |  |
|  | SRS | 0.04 | 296.86 | 0.02 | .981 |  |  |  |  |
|  | Cognitive Ability | -19.93 | 305.05 | -2.55 | .011* |  |  |  |  |
| **Model 4A** | | | | | | | | | |
|  |  |  |  |  |  | 0.01/ 0.01 | 32310.3 | 4.41 | .621 |
| Slope | Time | -20.17 | 1796.08 | -0.29 | .772 |  |  |  |  |
|  | Condition | 93.85 | 1790.73 | 0.35 | .727 |  |  |  |  |
|  | SRS | 1.03 | 1976.35 | 0.15 | .879 |  |  |  |  |
|  | Cognitive Ability | -19.80 | 304.84 | -2.53 | .012* |  |  |  |  |
|  | Time x Condition | -1.23 | 1793.90 | -0.01 | .990 |  |  |  |  |
|  | Time x SRS | 0.39 | 1797.30 | 0.16 | .875 |  |  |  |  |
|  | Condition x SRS | -8.63 | 1792.85 | -0.91 | .366 |  |  |  |  |
|  | Time x Condition x SRS | 1.89 | 1795.03 | 0.54 | .578 |  |  |  |  |
| **Model 3B** | | | | | | | | | |
|  |  |  |  |  |  | 0.00/ 0.01 | 32306.5 | 0.21 | .898 |
| Slope | Time | 15.25 | 1794.63 | 0.96 | .337 |  |  |  |  |
|  | Condition | -13.69 | 1793.20 | -0.39 | .699 |  |  |  |  |
|  | Support | -1.07 | 303.14 | -0.26 | .799 |  |  |  |  |
|  | Cognitive Ability | -19.78 | 306.87 | -2.54 | .011* |  |  |  |  |
| **Model 4B** | | | | | | | | | |
|  |  |  |  |  |  | 0.01/ 0.01 | 32308.8 | 5.92 | .433 |
| Slope | Time | 95.45 | 1793.94 | 1.49 | .136 |  |  |  |  |
|  | Condition | 71.11 | 1790.10 | 0.29 | .774 |  |  |  |  |
|  | Support | -21.83 | 1975.05 | -1.53 | .125 |  |  |  |  |
|  | Cognitive Ability | -19.54 | 306.65 | -2.51 | .012* |  |  |  |  |
|  | Time x Condition | -52.11 | 1792.79 | -0.58 | .565 |  |  |  |  |
|  | Time x Support | 9.10 | 1795.89 | 1.76 | .079 |  |  |  |  |
|  | Condition x Support | 17.92 | 1791.42 | 0.89 | .373 |  |  |  |  |
|  | Time x Condition x Support | -8.76 | 1794.30 | -1.19 | .233 |  |  |  |  |

Note. * p < .05 ** p < .01 *** p < .001. Slope indicates average learning on learning task trials. Time indicates time points 1 through 4 in the learning task. Condition indicates condition of the learning task (coded 0 = threat, 1 = control). SRS indicates social rejection sensitivity. Support indicates perceived social support. Cognitive ability was measured with the Raven’s Advanced Progressive Matrices. Believability indicates how believable participants found the study’s social evaluative threat. Models 3A and 4A included social rejection sensitivity as predictor, and models 3B and 4B included social support as predictor.

**Table S5**

*Models Investigating the Effects of Time, Condition, Social Rejection Sensitivity (Models 3/4A) and Social Support (Models 3/4B) on Reaction Time*

| Dependent Variable | Predictor Variable(s) | *b* | df | *t* | *p* | *R^2^m/R^2^c* | *AIC* | *Chi^2^* | *p_(chi2)_* |
| --- | --- | --- | --- | --- | --- | --- | --- | --- | --- |
| **Model 1** | | | | | | | | | |
|  |  |  |  |  |  | 0.01/0.20 | 32244.1 |  |  |
| RT | Time | -57.06 | 1747.04 | -3.88 | < .001*** |  |  |  |  |
|  | Believability | 0.50 | 251.99 | 0.54 | .592 |  |  |  |  |
| **Model 2** | | | | | | | | | |
|  |  |  |  |  |  | 0.01/0.20 | 32241.6 | 2.80 | .094 |
| RT | Time | -56.96 | 1747.07 | -3.88 | < .001*** |  |  |  |  |
|  | Condition | -54.80 | 1746.58 | -1.68 | .094 |  |  |  |  |
| **Model 3A** | | | | | | | | | |
|  |  |  |  |  |  | 0.01/0.20 | 32241.4 | 6.71 | .035* |
| RT | Time | -57.11 | 1746.90 | -3.89 | < .001*** |  |  |  |  |
|  | Condition | -54.70 | 1746.47 | -1.67 | .095 |  |  |  |  |
|  | SRS | 6.00 | 253.50 | 2.00 | .047 |  |  |  |  |
|  | Believability | 0.55 | 251.85 | 0.59 | .557 |  |  |  |  |
| **Model 4A** | | | | | | | | | |
|  |  |  |  |  |  | 0.01/0.20 | 32245.1 | 2.60 | .626 |
| RT | Time | -68.42 | 1748.19 | -1.06 | .288 |  |  |  |  |
|  | Condition | 146.04 | 1745.22 | 0.59 | .556 |  |  |  |  |
|  | SRS | 7.89 | 1892.51 | 1.18 | .237 |  |  |  |  |
|  | Time x Condition | -24.72 | 1747.06 | -0.27 | .786 |  |  |  |  |
|  | Time x SRS | 0.27 | 1749.13 | 0.12 | .907 |  |  |  |  |
|  | Condition x SRS | -8.29 | 1746.36 | -0.94 | .347 |  |  |  |  |
|  | Time x Condition x SRS | 1.23 | 1747.92 | 0.38 | .702 |  |  |  |  |
| **Model 3B** | | | | | | | | | |
|  |  |  |  |  |  | 0.01/0.20 | 32243.0 | 3.41 | .182 |
| RT | Time | -57.00 | 1746.94 | -3.88 | < .001*** |  |  |  |  |
|  | Condition | -54.79 | 1746.47 | -1.68 | .094 |  |  |  |  |
|  | Support | -4.99 | 254.99 | -0.78 | .436 |  |  |  |  |
| **Model 4B** | | | | | | | | | |
|  |  |  |  |  |  | 0.01/0.20 | 32245.3 | 9.14 | .166 |
| RT | Time | 17.59 | 1747.23 | 0.30 | .766 |  |  |  |  |
|  | Condition | 407.94 | 1744.71 | 1.78 | .075 |  |  |  |  |
|  | Support | -23.00 | 1889.13 | -1.63 | .103 |  |  |  |  |
|  | Time x Condition | -175.79 | 1746.61 | -2.10 | .036 |  |  |  |  |
|  | Time x Support | 6.80 | 1748.80 | 1.42 | .156 |  |  |  |  |
|  | Condition x Support | 41.78 | 1745.52 | 2.25 | .025 |  |  |  |  |
|  | Time x Condition x Support | -15.89 | 1747.90 | -2.34 | .019* |  |  |  |  |

Note. * p < .025 ** p < .01 *** p < .001. RT indicates average reaction time on correct trials of the learning task. Time indicates time points 1 through 4 in the learning task. Condition indicates condition of the learning task (coded 0 = threat, 1 = control). SRS indicates social rejection sensitivity^18^. Support indicates perceived social support^19^. Believability indicates how believable participants found the study’s social evaluative threat. Models 3A and 4A included social rejection sensitivity as predictor, and models 3B and 4B included social support as predictor.

**Table S6**

*Models Investigating the Effects of Time, Condition, Social Rejection Sensitivity (Models 3/4A) and Social Support (Models 3/4B) on Accuracy*

| Dependent Variable | Predictor Variable(s) | *b* | df | *t* | *p* | *R^2^m/R^2^c* | *AIC* | *Chi^2^* | *p_(chi2)_* |
| --- | --- | --- | --- | --- | --- | --- | --- | --- | --- |
| **Model 1** | | | | | | | | | |
|  |  |  |  |  |  | 0.02/0.56 | 8057.0 |  |  |
| Accuracy | Time | 0. 29 | 1740.00 | 9.34 | < .001*** |  |  |  |  |
| **Model 2** | | | | | | | | | |
|  |  |  |  |  |  | 0.02/0.56 | 8048.6 | 11.00 | < .001*** |
| Accuracy | Time | 0. 29 | 1740.00 | 9.36 | < .001*** |  |  |  |  |
|  | Condition | 0.23 | 1740.00 | 3.32 | < .001*** |  |  |  |  |
|  | Believability | -.00 | 249.10 | -1.19 | .235 |  |  |  |  |
| **Model 3A** | | | | | | | | | |
|  |  |  |  |  |  | 0.02/0.56 | 8049.3 | 0.69 | .408 |
| Accuracy | Time | 0.29 | 1739.91 | 9.36 | < .001*** |  |  |  |  |
|  | Condition | 0.23 | 1739.85 | 3.32 | < .001*** |  |  |  |  |
|  | SRS | -0.01 | 249.82 | -0.83 | .408 |  |  |  |  |
| **Model 4A** | | | | | | | | | |
|  |  |  |  |  |  | 0.03/0.56 | 8049.3 | 8.70 | .122 |
| Accuracy | Time | 0.07 | 1740.00 | 0.49 | .624 |  |  |  |  |
|  | Condition | -0.63 | 1739.00 | -1.19 | .235 |  |  |  |  |
|  | SRS | -0.04 | 898.70 | -2.31 | 0.021* |  |  |  |  |
|  | Time x Condition | 0.21 | 1740.00 | 1.06 | .288 |  |  |  |  |
|  | Time x SRS | 0.01 | 1741.00 | 2.01 | .045 |  |  |  |  |
|  | Condition x SRS | 0.04 | 1740.00 | 2.05 | .041 |  |  |  |  |
|  | Time x Condition x SRS | -.01 | 1740.00 | -1.48 | .138 |  |  |  |  |
| **Model 3B** | | | | | | | | | |
|  |  |  |  |  |  | 0.05/0.56 | 8041.2 | 9.56 | .002** |
| Accuracy | Time | 0.29 | 1740.00 | 9.37 | < .001*** |  |  |  |  |
|  | Condition | 0.23 | 1740.00 | 3.32 | < .001*** |  |  |  |  |
|  | Support | 0.08 | 250.60 | 3.09 | .002** |  |  |  |  |
|  | Believability | -0.00 | 249.50 | -1.11 | .267 |  |  |  |  |
| **Model 4B** | | | | | | | | | |
|  |  |  |  |  |  | 0.04/0.56 | 8041.8 | 6.62 | .157 |
| Accuracy | Time | 0.12 | 1740.55 | 0.97 | .335 |  |  |  |  |
|  | Condition | -0.04 | 1739.63 | -0.08 | .940 |  |  |  |  |
|  | Support | 0.14 | 928.63 | 3.62 | < .001*** |  |  |  |  |
|  | Time x Condition | 0.02 | 1740.35 | 0.09 | .926 |  |  |  |  |
|  | Time x Support | -0.02 | 1741.16 | -1.73 | .083 |  |  |  |  |
|  | Condition x Support | -0.04 | 1739.90 | -0.94 | .348 |  |  |  |  |
|  | Time x Condition x Support | 0.01 | 1740.86 | 0.49 | .625 |  |  |  |  |

Note. * p < .025 ** p < .01 *** p < .001. Accuracy indicates number of correct responses on the learning task (20 total responses for each time point). Time indicates time points 1 through 4 in the learning task. Condition indicates condition of the learning task (coded 0 = threat, 1 = control). SRS indicates social rejection sensitivity^18^. Support indicates perceived social support^19^. Models 3A and 4A included social rejection sensitivity as predictor, and models 3B and 4B included social support as predictor.

Supplementary Results for Hypothesis 2 Analyses

**Table S7**

*Models of the Effect of Depression on Mood*

| Dependent Variable | Predictor Variable(s) | *b* | df | *t* | *p* | *R^2^m/R^2^c* | *AIC* | *Chi^2^* | *P_(chi2)_* |
| --- | --- | --- | --- | --- | --- | --- | --- | --- | --- |
| **Model 1** | | | | | | | | | |
|  | | | | | | 0.06/0.66 | 4761.3 |  |  |
| Mood | Condition | 23.21 | 223.83 | 6.97 | <.001*** |  |  |  |  |
|  | Believability | -0.30 | 219.87 | -2.52 | .012* |  |  |  |  |
| **Model 2** | | | | | | | | | |
|  | | | | | | 0.04/0.66 | 4766.9 | 0.69 | .405 |
| Mood | Condition | 23.21 | 223.72 | 6.96 | <.001*** |  |  |  |  |
|  | Depression | 0.31 | 219.80 | 0.83 | .406 |  |  |  |  |
| **Model 3** | | | | | | | | | |
|  |  |  |  |  |  | 0.04/0.66 | 4766.8 | 2.75 | .253 |
| Mood | Condition | 15.98 | 223.72 | 2.65 | .009** |  |  |  |  |
|  | Depression | 0.06 | 310.14 | 0.15 | .879 |  |  |  |  |
|  | Condition x Depression | 0.49 | 223.72 | 1.44 | .152 |  |  |  |  |

Note. * p < .05 ** p < .01 *** p < .001. Mood is operationalised as the sum of anxiety (out of 100), stress (out of 100), and reverse-coded pleasantness (out of 100) measures, with higher scores indicating greater negative mood. Condition is modelled as threat (baseline mood minus post-threat mood) versus control (baseline mood minus post-control mood). Depression reflects levels of depressive symptoms measured with the depression subscale of the short Depression, Anxiety and Stress Scale^5^. Believability indicates how believable participants found the study’s social evaluative threat.

**Table S8**

*Models of the Effect of Mental Health on Mood*

| Dependent Variable | Predictor Variable(s) | *b* | df | *t* | *p* | *R^2^m/R^2^c* | *AIC* | *Chi^2^* | *p_(chi2)_* |
| --- | --- | --- | --- | --- | --- | --- | --- | --- | --- |
| **Model 1** | | | | | | | | | |
|  |  |  |  |  |  | 0.06/0.66 | 4761.3 |  |  |
| Mood | Condition | 23.21 | 223.83 | 6.97 | <.000*** |  |  |  |  |
|  | Believability | -0.30 | 219.87 | -2.52 | .012* |  |  |  |  |
| **Model 2** | | | | | | | | | |
|  |  |  |  |  |  | 0.04/0.66 | 4767.4 | 0.23 | .634 |
| Mood | Condition | 23.21 | 223.74 | 6.96 | <.000*** |  |  |  |  |
|  | Health | 0.63 | 218.56 | 0.48 | .635 |  |  |  |  |
| **Model 3** | | | | | | | | | |
|  |  |  |  |  |  | 0.04/0.66 | 4766.7 | 2.90 | .234 |
| Mood | Condition | 23.81 | 223.75 | 7.14 | <.000*** |  |  |  |  |
|  | Health | -0.36 | 306.93 | -0.25 | .803 |  |  |  |  |
|  | Condition x Health | 1.99 | 223.75 | 1.64 | .102 |  |  |  |  |

Note. * p < .05 ** p < .01 *** p < .001. Mood is operationalised as the sum of anxiety (out of 100), stress (out of 100), and reverse-coded pleasantness (out of 100) measures, with higher scores indicating greater negative mood. Condition is modelled as threat (baseline mood minus post-threat mood) versus control (baseline mood minus post-control mood). Health indicates composite mental health score (standardized and summed WEMWS, SDQ and DASS-21 scores). Believability indicates how believable participants found the study’s social evaluative threat.

**Table S9**

*Models of the Effect of Depression (Models 2/3A) and Mental Health (Models 2/3B) on Learning*

| Dependent Variable | Predictor Variable(s) | *b* | df | *t* | *p* | *R^2^m/R^2^c* | *AIC* | *Chi^2^* | *p_(chi2)_* |
| --- | --- | --- | --- | --- | --- | --- | --- | --- | --- |
| **Model 1** | | | | | | | | | |
|  |  |  |  |  |  | 0.00/0.05 | 6880.3 |  |  |
| Slope | Condition | 111.13 | 292.29 | 1.40 | .162 |  |  |  |  |
|  | Cognitive ability | 8.81 | 287.65 | 0.46 | .648 |  |  |  |  |
| **Model 2A** | | | | | | | | | |
|  |  |  |  |  |  | 0.02/0.05 | 6879.0 | 4.49 | .034* |
| Slope | Condition | 109.96 | 292.93 | 1.39 | .166 |  |  |  |  |
|  | Depression | 9.59 | 288.31 | 2.19 | .029* |  |  |  |  |
|  | Cognitive ability | 17.18 | 284.83 | 0.87 | .384 |  |  |  |  |
|  | Believability | -1.20 | 290.81 | -0.87 | .385 |  |  |  |  |
| **Model 3A** | | | | | | | | | |
|  |  |  |  |  |  | 0.02/0.05 | 6879.7 | 0.12 | .734 |
| Slope | Condition | 68.30 | 296.13 | 0.47 | .639 |  |  |  |  |
|  | Depression | 7.90 | 420.91 | 1.30 | .195 |  |  |  |  |
|  | Cognitive ability | 18.96 | 286.35 | 0.97 | .335 |  |  |  |  |
|  | Condition x Depression | 2.80 | 294.64 | 0.34 | .734 |  |  |  |  |
| **Model 2B** | | | | | | | | | |
|  |  |  |  |  |  | 0.02/0.04 | 6879.8 | 3.97 | .046* |
| Slope | Condition | 109.81 | 289.76 | 1.38 | .168 |  |  |  |  |
|  | Health | 31.27 | 269.41 | 2.03 | .044 |  |  |  |  |
|  | Cognitive ability | 16.31 | 282.56 | 0.83 | .408 |  |  |  |  |
|  | Believability | -1.15 | 286.76 | -0.84 | .404 |  |  |  |  |
| **Model 3B** | | | | | | | | | |
|  |  |  |  |  |  | 0.02/0.04 | 6880.2 | 4.10 | .129 |
| Slope | Condition | 104.27 | 289.66 | 1.30 | .193 |  |  |  |  |
|  | Health | 38.82 | 419.62 | 1.801 | .071 |  |  |  |  |
|  | Cognitive ability | 18.02 | 283.82 | 0.92 | .358 |  |  |  |  |
|  | Condition x Health | -16.12 | 289.21 | -0.55 | .581 |  |  |  |  |

Note. * p < .05 ** p < .01 *** p < .001. Slope indicates average slope on time point 4 in the learning task minus average slope on time point 1 in the learning task. Condition indicates condition of the learning task (coded 0 = threat, 1 = control). Depression indicates depressive symptoms^5^. Health indicates composite mental health score (standardized and summed WEMWS, SDQ and DASS-21 scores). Cognitive ability was measured with the Raven’s Advanced Progressive Matrices. Believability indicates how believable participants found the study’s social evaluative threat. Models 2A and 3A included depression as predictor, and models 2B and 3B included mental health as predictor.

Supplementary Results for Hypothesis 3 Analyses

Believability ratings were included as a covariate for all analyses. Cognitive ability was included as a covariate for all learning analyses. It is important to note that this was a cross-sectional study, so any significant associations between factors do not imply causal or temporal mediation.

Despite the absence of a significant effect of pre-existing depressive symptoms, social rejection sensitivity partially accounted for variance in the relationship between depressive symptoms and mood (operationalised as changes in reported mood from baseline to post-threat, and from baseline to post-control; *b* = -0.01, *SE* = 0.00, *z* = -2.40, *p* = 0.016), such that individuals with higher depressive symptoms also reported higher social rejection sensitivity and an increase in negative mood during the experiment.

As hypothesised, social rejection sensitivity partially accounted for variance in the relationship between mental health and mood (*b* = -0.03, *SE* = 0.01, *z* = -2.24, *p* = .025), such that individuals with increased negative mental health symptoms also reported increased SRS and increased negative mood.

References

1. Ratcliff, R. Methods for dealing with reaction time outliers. *Psychological Bulletin* **114**, 510–532 (1993).

2. Tennant, R. *et al.* The Warwick-Edinburgh Mental Well-being Scale (WEMWBS): development and UK validation. *Health and Quality of Life Outcomes* **5**, 63 (2007).

3. Goodman, R. The Strengths and Difficulties Questionnaire: a research note. *Journal of Child Psychology and Psychiatry* **38**, 581–586 (1997).

4. He, J. P., Burstein, M., Schmitz, A. & Merikangas, K. R. The Strengths and Difficulties Questionnaire (SDQ): the factor structure and scale validation in U.S. adolescents. *Journal of Abnormal Child Psychology* **41**, 583–95 (2013).

5. Lovibond, P. F. & Lovibond, S. H. *Manual for the Depression Anxiety Stress Scales*. (Psychology Foundation of Australia, 1995).

6. Chierchia, G. *et al.* The matrix reasoning item bank (MaRs-IB): novel, open-access abstract reasoning items for adolescents and adults. *Royal Society Open Science* **6**, 190232 (2019).

7. Australian Bureau of Statistics. Census of Population and Housing: Reflecting Australia - Stories from the Census, 2016. **Catalogue No. 2071.0**, (2017).

8. Raven, J. C. *Guide to the standard progressive matrices: Sets A, B, C, D and E*. (HK Lewis & Co. Ltd, 1960).

9. Schweizer, S. *et al.* Protocol for an app-based affective control training for adolescents: proof-of-principle double-blind randomized controlled trial. *Wellcome Open Research* **4**, 91 (2019).

10. Lezak, M. D. *Neuropsychological assessment*. (Oxford University Press, 1995).

11. Dan-Glauser, E. S. & Scherer, K. R. The Geneva affective picture database (GAPED): A new 730-picture database focusing on valence and normative significance. *Behavior Research Methods* **43**, 468–477 (2011).

12. Bates, D., Mächler, M., Bolker, B. & Walker, S. Fitting linear mixed-effects models using lme4. *Journal of Statistical Software* **67**, 1–48 (2015).

13. Lenth, R. V. Using lsmeans. *Journal of Statistical Software* **69**, 1–33 (2017).

14. Wickham, H., François, R., Henry, L., Müller, K., & RStudio. dplyr: A Grammar of Data Manipulation. (2022).

15. Zhang, D. rsq: R-Squared and Related Measure. (2021).

16. Tingley, D., Yamamoto, T., Hirose, K., Keele, L. & Imai, K. Mediation: R package for causal mediation analysis. *Journal of Statistical Software* **59**, 1–38 (2014).

17. Rosseel, Y. Lavaan: An R package for structural equation modeling and more. Version 0.5–12 (BETA). *Journal of statistical software* **48**, 1–36 (2012).

18. Andrews, J. L., Khin, A. C., Crayn, T., Humphreys, K. & Schweizer, S. Measuring the Role of Social Rejection Sensitivity in Depressive Symptoms in the Digital Age: The Online and Offline Social Sensitivity Scale. (2021).

19. Schuster, T. L., Kessler, R. C. & Aseltine, R. H. Supportive Interactions, Negative Interactions, and Depressed Mood. *American Journal of Community Psychology* **18**, 423–438 (1990).
